# Influenza Vaccination Uptake among the Working Age Population of Japan: Results from a National Cross-Sectional Survey

**DOI:** 10.1371/journal.pone.0059272

**Published:** 2013-03-12

**Authors:** Koji Wada, Derek R. Smith

**Affiliations:** 1 Department of Public Health, Kitasato University School of Medicine, Sagamihara, Japan; 2 School of Health Sciences, Faculty of Health, University of Newcastle, Brush Road, Ourimbah, Australia; Alberta Provincial Laboratory for Public Health/ University of Alberta, Canada

## Abstract

**Background:**

Influenza vaccination rates among Japanese people of working age (20–69 years) is currently suboptimal, and the reasons for this have not been clearly elucidated. This study examined factors associated with vaccination intention among the working age population in Japan during September 2011, one-month prior to influenza vaccination becoming available.

**Methodology/Principal Findings:**

A web-based survey of intention to be vaccinated against influenza in the coming season was undertaken among 3,129 Japanese aged 20 to 69 years. Multinomial logistic regression analysis was used to explore the associations between vaccination intent and other variables. Influenza vaccination intent was associated with having been vaccinated in the previous year (Odds Ratio (OR): 3.81; 95% Confidence Interval (CI): 3.75–3.86), the number of children per household (one compared with zero; OR: 1.37; 95%CI: 1.11–1.65), and household income ($50,000 to <$100,000 compared with $0 to <$50,000; OR: 1.30; 95%CI: 1.07–1.54). Smoking was inversely associated with influenza vaccine uptake (current smokers compared with non-smokers; OR: 0.79; 95%CI: 0.61–0.98). A history of either the survey respondent or a household member having being medically diagnosed with influenza in the previous year was not statistically associated with future influenza vaccination intent.

**Conclusions/Significance:**

Overall, this suggests that intention to be vaccinated among working age Japanese is associated with a past history of influenza vaccination, having children, and the household's income. As such, consideration of these factors should now form the cornerstone of strategies to encourage increased uptake of vaccination against influenza in future years.

## Introduction

Influenza vaccination coverage in adults is relatively low in many countries, and low coverage was also observed during the H1N1 influenza pandemic of 2009 [1], [2]. The Japanese government procured influenza vaccine for the entire population in 2009, but more than 99.7 million doses of the stockpiled vaccine (81.8% of the total ordered) ended up not being used [3]. The reasons for this low coverage are unknown, although possible reasons may relate to concerns of vaccine safety [4] and efficacy [5] – concerns that have been voiced for more than 20 years by an anti-vaccination lobby in Japan claiming that influenza vaccine is of limited efficacy and causes side effects [6]. Other reasons for suboptimal influenza vaccination coverage might relate to limited motivation, insufficient income or not living in households considered to be at risk [7], [8].

Influenza vaccination is recommended for individuals over 6 months of age by the United States Centers for Disease Control and Prevention [9]. On the other hand, in Japan, the working age population has not been recommended to receive influenza vaccine, and influenza vaccination coverage is relatively low in this group (around 25%) [10]. As such, predictors of influenza vaccination intention should be addressed to build strategies to encourage yearly influenza vaccination.

Predictors of influenza vaccination among the working age Japanese population were previously investigated in a study of 428 adults that found that vaccinated individuals tended to have underlying diseases, perceived themselves as being susceptible to influenza, and found the vaccines to be affordable [11]. Other predictors such as demographic characteristics, a prior history of vaccination, and history of influenza infection for survey respondents or household members, have not been well studied and therefore need to be investigated in a larger sample of Japanese people. As such, this study aimed to determine the factors associated with the intention to be vaccinated against influenza in September 2011 (one-month before the influenza vaccine became available) among the working age population of Japan.

## Materials and Methods

### Ethics statement

This study was approved by the Human Research Committee at the Kitasato University School of Medicine.

### Data collection

This study sought to recruit a total of 3,000 Japanese individuals aged 20 to 69 years who had been registered by a web-based survey company. Registrants were individuals interested in participating in a survey that provided financial incentives. In September 2011, the survey company randomly selected persons from this list and invited them to participate in the current study. Recruitment was intended to cease when the number of participants had reached the target (3,000), although 3,129 persons eventually agreed to participate from a total of 7,937 individuals initially contacted. Participants were classified into five groups by age range: 20–29, 30–39, 40–49, 50–59 and 60-69 years. Individuals who agreed to participate were then directed to complete an anonymous online questionnaire.

Questions included basic demographic information (including age, gender, number of children living in the household, household income in Yen [1US$ = 80Yen at the time], and smoking status), influenza vaccination status in the previous year, and whether the participant or any member of their household had been medically diagnosed with influenza in the previous year. The survey also enquired about other hygiene practices that had been associated with vaccination in a previous study (wearing a face mask in public and hand washing); as these factors were to be used during multivariate analysis of data [12].

To determine vaccination intent, the question “Do you intend to receive an influenza vaccine in this coming influenza season?” was asked, with possible answers of “Yes”, “Not decided yet”, or “No”. To determine vaccination status in the previous year, we asked: “Did you receive an influenza vaccine in the previous year?” with possible answers of “Yes” or “No”. We also enquired about their prior history of influenza infection with the following two questions: “During the period from October 2010 to March 2011, were you medically diagnosed with influenza?” and “During the period from October 2010 to March 2011, was a member of your family who lives with you medically diagnosed with influenza?”

### Statistical analysis

We conducted univariate analysis using Pearson's chi-squared test to examine the potential relationships between vaccination intent and other demographic variables. Multinomial logistic regression analyses was then used to explore possible associations between predictor variables and the outcome of vaccination intent in the coming year. The three outcome categories were as follows: “I will not receive a vaccine” (referent), “I am not decided yet”, and “I am intending to receive a vaccine”. All analyses were performed using IBM SPSS Statistics 19, with statistical significance being set at p<0.05.

## Results

A total of 3,129 persons, including 1,572 men and 1,557 women, participated in the study. Table 1 indicates the characteristics of participants. A total of 25.4% expressed an intention to be vaccinated in the coming influenza season and 27.7% were undecided, even though the survey was conducted only 1 month before the start of influenza vaccine becoming available. In the previous influenza season, 7.3% of respondents had been medically diagnosed with influenza and 15.3% of respondents reported having had a household member who was diagnosed with influenza.

**Table 1:** Participant Characteristics.

	n	(%)
	(3,129)	
Age (years)		
20–29	510	(16.3)
30–39	659	(21.1)
40–49	647	(20.7)
50–59	601	(19.2)
60–69	712	(22.8)
Gender		
Male	1,572	(50.2)
Intention to be vaccinated in the coming season		
Yes, I intend to be vaccinated	796	(25.4)
Not yet decided	867	(27.7)
Will not be vaccinated	1,466	(46.9)
Influenza vaccination in the previous year		
Vaccinated	809	(25.9)
Not vaccinated	2,320	(74.1)
Number of children in household		
None	1,821	(58.2)
One	645	(20.6)
Two or more	663	(21.2)
Yearly household income (USD)		
$0 to <$50,000	1,213	(38.8)
$50,000 to <$100,000	1,326	(42.4)
$100,000+	590	(18.9)
Smoking		
Current smoker	663	(21.1)
Former smoker	700	(22.4)
Never smoked	1,766	(56.4)
Medically diagnosed with influenza in the previous year	229	(7.3)
Household member medically diagnosed with influenza in the previous year (n = 2,899; excluding 230 who did not have household members)	444	(15.3)

Table 2 shows associations between vaccination intent and other study variables. A strong association was seen between prior vaccination status and intention to be vaccinated in the coming season. In fact, 72.7% of respondents who were vaccinated in the previous year also intended to be vaccinated in the coming season, while only 9.0% of unvaccinated respondents in the previous year expressed vaccination intent for the coming season. Thirty-three percent of respondents with at least one child in their household intended to be vaccinated. Intention to be vaccinated against influenza was also related to household income, with respondents from higher-income households more likely to express an intention for future vaccination. A significantly higher number of participants who had been diagnosed with influenza in the previous year intended to be vaccinated in the coming period. Similarly, participants with household members who had been diagnosed with influenza were more likely to express an intention to be vaccinated in future when compared to household members without a previous history of influenza diagnosis.

**Table 2:** Associations between intention to receive influenza vaccine in the coming influenza season and other study variables (n = 3,129).

	Intend to be vaccinated in the coming season		Not yet decided		Will not be vaccinated		
	n = 796	(%)	n = 867	(%)	n = 1466	(%)	*P* value^a^
Age (years)							
20–29	148	(29.0)	195	(38.2)	167	(32.7)	<0.001
30–39	169	(25.6)	175	(26.6)	315	(47.8)	
40–49	151	(23.3)	174	(26.6)	322	(49.8)	
50–59	156	(26.0)	162	(27.0)	283	(47.1)	
60–69	172	(24.2)	161	(22.6)	379	(53.2)	
Gender							
Male	431	(27.4)	457	(29.1)	684	(43.5)	<0.001
Female	365	(23.4)	410	(26.3)	782	(50.2)	
Influenza vaccination in the previous year							
Vaccinated	588	(72.7)	158	(19.5)	63	(7.8)	<0.001
Not vaccinated	208	(9.0)	709	(30.6)	1403	(60.5)	
Number of children in household							
None	383	(21.0)	532	(29.2)	906	(49.8)	<0.001
One	213	(33.0)	173	(26.8)	259	(40.2)	
Two or more	200	(30.2)	162	(24.4)	301	(45.4)	
Household income (USD)							
$0 to <$50,000	240	(19.8)	366	(30.2)	607	(50.0)	<0.001
$50,000 to <$100,000	369	(27.8)	369	(27.8)	588	(44.3)	
$100,000+	187	(31.7)	132	(22.4)	271	(45.9)	
Smoking							
Current smoker	134	(20.2)	174	(26.2)	355	(53.5)	<0.001
Former smoker	169	(24.1)	202	(28.9)	329	(47.0)	
Never smoked	493	(27.9)	491	(27.8)	782	(44.3)	
Medically diagnosed with influenza in the previous year							
Yes	89	(38.9)	73	(31.9)	67	(29.3)	<0.001
No	707	(22.8)	794	(25.6)	1599	(51.6)	
Household member diagnosed with influenza in the previous year							
Yes	162	(36.5)	104	(23.4)	178	(40.1)	<0.001
No	634	(23.6)	763	(28.4)	1288	(48.0)	

a Statistical differences by intention to receive influenza vaccine with Chi-square test.

Table 3 details the results of multinomial logistic regression analysis when considering intention to be vaccinated and other study variables. Compared with participants who had not been vaccinated in the previous year, respondents who *were* vaccinated had a higher odds of vaccination intent in the coming season (Odds Ratio (OR): 3.81; 95% Confidence Interval (CI): 3.75–3.86). Similarly, respondents who were vaccinated in the previous year were more likely to have not yet decided whether or not they would be vaccinated in the coming season (OR: 2.41; 95%CI: 2.14–2.66). The number of children per household (one compared with zero; OR: 1.37; 95%CI: 1.11–1.65) and household income ($50,000 to $100,000 compared with $0 to <$50,000; OR: 1.30; 95%CI: 1.07–1.54) were both positively associated with vaccination intent in the coming season. Current smoking status (OR: 0.79; 95%CI: 0.61–0.98) was negatively associated with future intention to be vaccinated. A history of prior influenza infection for either the survey participant or any of their household members was not significantly associated with intention to receive influenza vaccine.

**Table 3:** Multinomial logistic regression analysis of associations between study variables and intention to be vaccinated in the coming influenza season (reference category: will not be vaccinated).

	Intend to be vaccinated in the coming season				Not yet decided			
	Crude		Multivariate^a^		Crude		Multivariate^a^	
	OR	(95% CI)	OR	(95% CI)	OR	(95% CI)	OR	(95% CI)
Influenza vaccination in the previous year								
Vaccinated versus unvaccinated (ref)	3.82	(3.76–3.86)	3.81	(3.75–3.86)	2.44	(2.17–2.67)	2.41	(2.14–2.66)
Number of children in household								
None	ref		ref		ref		ref	
One	1.61	(1.41–1.83)	1.37	(1.11–1.65)	1.13	(0.97–1.31)	1.11	(0.94–1.30)
Two or more	1.41	(1.22–1.61)	1.21	(0.96–1.50)	0.97	(0.82–1.13)	0.97	(0.81–1.16)
Household income (US $)								
0 to <50,000	ref		ref		ref		ref	
50,000 to <100,000	1.44	(1.27–1.63)	1.30	(1.07–1.54)	1.08	(0.94–1.22)	1.04	(0.90–1.19)
100,000+	1.53	(1.31–1.76)	1.17	(0.92–1.47)	0.90	(0.74–1.07)	0.83	(0.68–1.01)
Smoking (versus not smoking)								
Current smoker	0.70	(0.58–0.84)	0.79	(0.61–0.98)	0.87	(0.74–1.02)	0.91	(0.76–1.07)
Former smoker	0.89	(0.75–1.05)	0.81	(0.64–1.02)	1.02	(0.87–1.18)	1.01	(0.86–1.18)
Never smoked	ref		ref		ref		ref	
Medically diagnosed with influenza in the previous year	1.82	(1.51–2.14)	1.08	(0.73–1.53)	1.49	(1.21–1.80)	1.20	(0.89–1.55)
Household member diagnosed with influenza in the previous year	1.56	(1.34–1.79)	0.74	(0.51–1.04)	1.02	(0.84–1.22)	0.82	(0.62–1.07)

a Adjusted for age and gender OR: odds ratio, CI: confidence interval, ref: referent.

a Multivariate model: adjusted for all independent variables, including hand washing and wearing a facemask in public.

## Discussion

The current study investigated predictors associated with intent to receive influenza vaccine among the working age population of Japan. The official Japanese influenza vaccine provision begins in October every year, and as such, our study was undertaken in September to determine the participants' intentions immediately prior to the national vaccine provision. We found that influenza vaccination in the previous year, the number of children per household, and household income were all associated with an intention to receive influenza vaccination. A prior diagnosis of influenza infection in the previous year was not associated with vaccination intent, although current smoking status was generally associated with a lower influenza vaccine uptake.

Some of our current findings are consistent with previous research, with various studies demonstrating that a history of influenza vaccination is a strong predictor of vaccination in the following year, in Japan as well as some other countries [11], [13]–[15]. Vaccinated individuals appear to have confidence in the safety and effectiveness of influenza vaccines, and this phenomenon is not limited to Japan. A recent study from Turkey, for example, found that the leading factor negatively influencing vaccine uptake was a disbelief in the vaccine's effectiveness [16]. Interestingly, 30.6% of respondents in the current study who were not vaccinated in the previous year had not yet decided if they would be vaccinated in the current year. Such a finding suggests that this group may be an ideal target to help increase vaccination uptake in Japan, as elsewhere.

The number of children per household was an important predictor of vaccination intent for working age adults in the current study. When compared with respondents with no children, there were significant associations between households with one child and their intention to be vaccinated, as well as a weak association between households with two or more children and their vaccination intent. It is well-known that children can be vulnerable to influenza, being at an increased risk of infection from schools and nursery schools, and also being at greater risk of developing a more severe illness if the disease is contracted [17]. Similarly, working age populations with children are also at risk of contracting influenza if their children are infected [18], [19]. For these reasons, individuals with children would likely have additional motivation to prevent influenza infection in themselves and their children, compared with people living without children. Despite this fact, however, not all studies have demonstrated such a trend. For example, a recent investigation of parents from the United Kingdom [20] found that while 61% would accept influenza vaccination for their children, the most common reasons for declining were concerns about safety and potential side effects.

Cost may also contribute to low influenza vaccination coverage rates in Japan as elsewhere, given that in Japan, people of working age must pay for influenza vaccination themselves. During the 2009 influenza pandemic, for example, one dose of influenza vaccine cost 3,600 yen for adults (around US $45) in this country [11]. Compared with other countries, this may be seen as relatively expensive [21], and as a result, individuals with limited income may simply be unable to afford the vaccine [7]. As a result, financial support for the working age population and/or subsidizing vaccination may be one strategy to help improve this situation in Japan. Offering free influenza vaccination days in Japan's Municipal Health Centers (MHC), for example, might help raise coverage rates among those unwilling or unable to pay for influenza vaccines. MHCs represent an important component of the national public health system in Japan, and already perform a variety of preventive services [22]. As such, adding influenza vaccination days at MHCs is a feasible option to increase influenza vaccination coverage. Further benefits might also arise from having designated “influenza vaccination days” at MHCs in Japan, not the least being increased public awareness as well as providing healthy role modeling behavior for children – similar to current, routine vaccination schedules.

The current study revealed a statistically significant relationship between some aspects of vaccination status and tobacco use. This is consistent with some previous research which revealed that smokers are unlikely to receive influenza vaccine [23], even though they have a high risk of influenza virus infection [24] and chronic obstructive pulmonary disease (COPD). Patients with COPD are strongly encouraged to be vaccinated against influenza [25]. Such a result is not entirely surprising however, as smokers tend to have mixture of unhealthy behaviors; including lower vegetable intake and higher alcohol consumption when compared to non-smokers [26]. As a result, any interventions to increase influenza vaccination uptake among smokers will clearly need to consider these issues in their design.

Interestingly, the current study revealed that previous influenza infection for survey participants and their household members was not significantly associated with an intention to receive influenza vaccine. To our knowledge, no similar studies have investigated the association between history of infection and future intention to be vaccinated. We found that among 805 participants who had been vaccinated in the previous year, 111 were diagnosed with influenza virus infection in the previous influenza season. Despite this fact, only 13.5% of them decided not to receive influenza vaccination in the coming season. On the other hand, 123 individuals out of 2,324 who did not receive a vaccine in the previous year had been diagnosed with influenza in the previous year. Of these 2,324, only 15.4% intended to be vaccinated in the coming vaccination provision. As such, this finding suggests that influenza infection itself, might not play a large role in determining future influenza vaccination uptake.

Limitations of the current study include the fact that the study population was recruited through a web-based survey company, and it might be expected that individuals who can access the internet can also seek information more easily and tend to take healthy behaviors more seriously [27]. Such factors will need to be investigated in future research. In addition, while the current study investigated intention to receive influenza vaccine, participants were not followed up and as such, we were unable to determine if participants subsequently received the vaccination or not. This may be a relevant consideration for future investigations, as a previous study by Yi and colleagues [11], for example, found that while 57% of people were willing to be vaccinated, only 12.1% actually ended up doing so. A result which suggests that individuals who state a willingness to be vaccinated at one point in time, might later change their mind. Future studies would do well to incorporate intervention strategies to increase motivation to receive influenza vaccine in such groups.

## Conclusion

Overall, this study found that among Japanese people of working age, intention to be vaccinated against influenza is associated with a prior history of influenza vaccination, number of children, and household income. These factors should form the cornerstone of strategies to encourage higher vaccination rates among working age populations in Japan, as elsewhere.
